# Whole-Body Distribution of Leukemia and Functional Total Marrow Irradiation Based on FLT-PET and Dual-Energy CT

**DOI:** 10.1177/1536012117732203

**Published:** 2017-09-26

**Authors:** Taiki Magome, Jerry Froelich, Shernan G. Holtan, Yutaka Takahashi, Michael R. Verneris, Keenan Brown, Kathryn Dusenbery, Jeffrey Wong, Susanta K. Hui

**Affiliations:** 1 Department of Radiological Sciences, Faculty of Health Sciences, Komazawa University, Tokyo, Japan; 2 Masonic Cancer Center, University of Minnesota, Minneapolis, MN, USA; 3 Department of Radiology, University of Minnesota, Minneapolis, MN, USA; 4 Blood and Marrow Transplant Program, University of Minnesota, Minneapolis, MN, USA; 5 Department of Radiation Oncology, Osaka University, Osaka, Japan; 6 Mindways Software Inc, Austin, TX, USA; 7 Department of Therapeutic Radiology, University of Minnesota, Minneapolis, MN, USA; 8 Department of Radiation Oncology, Beckman Research Institute, City of Hope, Duarte, CA, USA

**Keywords:** leukemia, FLT-PET, dual-energy CT, red marrow, yellow marrow, functional total marrow irradiation

## Abstract

This report describes a multimodal whole-body 3′-deoxy-3′[(18)F]-fluorothymidine positron emission tomography (FLT-PET) and dual-energy computed tomography (DECT) method to identify leukemia distribution within the bone marrow environment (BME) and to develop disease- and/or BME-specific radiation strategies. A control participant and a newly diagnosed patient with acute myeloid leukemia prior to induction chemotherapy were scanned with FLT-PET and DECT. The red marrow (RM) and yellow marrow (YM) of the BME were segmented from DECT using a basis material decomposition method. Functional total marrow irradiation (fTMI) treatment planning simulations were performed combining FLT-PET and DECT imaging to differentially target irradiation to the leukemia niche and the rest of the skeleton. Leukemia colonized both RM and YM regions, adheres to the cortical bone in the spine, and has enhanced activity in the proximal/distal femur, suggesting a potential association of leukemia with the BME. The planning target volume was reduced significantly in fTMI compared with conventional TMI. The dose to active disease (standardized uptake value >4) was increased by 2-fold, while maintaining doses to critical organs similar to those in conventional TMI. In conclusion, a hybrid system of functional–anatomical–physiological imaging can identify the spatial distribution of leukemia and will be useful to both help understand the leukemia niche and develop targeted radiation strategies.

## Introduction

Total marrow irradiation (TMI) with helical tomotherapy or volumetric-modulated arc therapy is a sophisticated technique for conditioning before hematopoietic cell transplantation.^1-3^ Total marrow irradiation focuses radiation to the entire skeletal anatomy albeit with the simplified premise that hematologic disease is distributed homogeneously in the bone marrow (BM). However, there is limited knowledge about the biological target and its spatial distribution, limiting the possibility of biological targeting rather than anatomical targeting. Furthermore, preclinical studies indicate the structural and functional heterogeneity of BM^4,5^ and the potential role of the local bone marrow environment (BME) in leukemia resistance.^6-9^ Despite the growing appreciation of the BME in preclinical systems, application at the clinical level has remained challenging. Bone marrow biopsies performed for diagnosis and prognostication provide valuable details regarding marrow composition and molecular profiles of malignant cells. However, a BM biopsy is an invasive procedure and is limited to specific anatomic regions, which may or may not reflect overall disease burden, and it lacks 3-dimensional resolution. It may be valuable to supplement diagnostic information from a BM biopsy with an assessment that considers the whole body (WB) to more fully document disease burden and develop a personalized approach to treating the leukemia niche.

We recently reported on the use of WB dual-energy computed tomography (DECT) to reveal a skeletal-wide heterogeneity within red marrow (RM) and yellow marrow (YM) composition, and the TMI irradiation technique was proposed for targeting specific BME regions, offering a significant dose reduction to critical organs.^10^ Furthermore, by use of 3′-deoxy-3′[(18)F]-fluorothymidine positron emission tomography (FLT-PET) imaging, the heterogeneous nature of acute myelogenous leukemia (AML) in the skeletal system was suggested.^11^ We now present a novel hybrid WB FLT-PET-DECT imaging strategy to identify the spatial distribution of leukemia and its association with the BME. We further show how hybrid imaging combined with intensity-modulated radiation could facilitate molecular image-guided functional marrow irradiation, consequently allowing further dose escalation.

## Materials and Methods

### Data Acquisition

After institutional review board approval, a control participant (full recovery patient, received total body irradiation 13.2 Gy, imaged at day 100 posttransplant) and a participant with newly diagnosed AML (female, 80% blasts measured by marrow aspirate) were imaged with FLT-PET and DECT (Biograph mCT; Siemens, Erlangen, Germany). At 60 minutes post-FLT (∼185 MBq) intravenous (IV) injection, WB static imaging was acquired. Acquisition time was 3 minutes per bed position. The DECT acquisitions (140 and 80 kVp energy, 5-mm slice thickness, 1.37-mm pixel size) were performed, followed by PET acquisition (5-mm slice thickness, 3.18-mm pixel size).

### Calculation of FLT-Avid Regions

The FLT-avid region was defined by standardized uptake value (SUV) as follows:

1 $$\text{SUV}=\frac{U}{D/w^{\prime }},$$

where *U* is the tissue uptake activity (Bq/g), *w* is the patient’s body weight (g), and *D* is the injected dose at the time of administration (Bq).

### Calculation of RM and YM Regions Based on DECT

The RM and YM were segmented from DECT based on a basis material decomposition by using QCT Pro (Mindways Software, Austin, Texas), with details reported previously.^10,12^ The volumetric overlap between FLT-avid and marrow regions was evaluated by Dice similarity coefficient (DSC).^13^ Binary PET images were created for the calculation of DSCs by changing the SUV threshold value. The DSC value ranges from 0 (noncorrespondence) to 1 (complete overlap).

### Simulation of Functional Marrow Irradiation

For TMI, the entire skeleton (including both RM and YM) was used as a clinical target volume (CTV), and the planning target volume (PTV) was generated with 5 mm margin to CTV. For differential targeted radiation, simulation was separated into 2 groups. Category I: Dose optimization to disease and BM, representing TMI and biological targeting using (i) FLT-based TMI, 18 Gy irradiation to FLT-avid regions and 12 Gy to the remaining skeleton, and (ii) DECT-based TMI, higher dose (18 Gy) irradiation to the functional marrow (ie, RM) detected by DECT and a lower dose (12 Gy) to rest of the skeletal system. Category II: Further dose escalation in PET-positive active disease. The details of treatment planning were reported previously.^10^

## Results and Discussion

**Figure 1.**
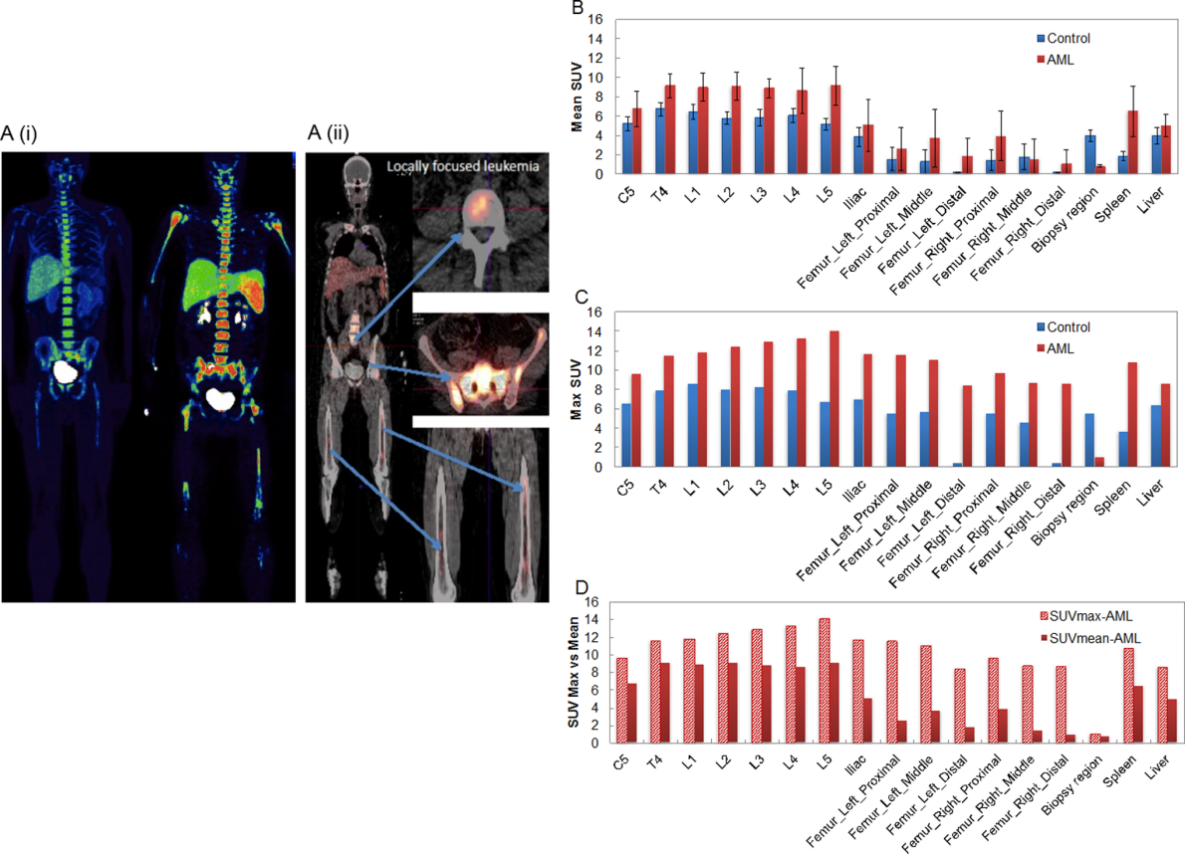
A, (i) Whole-body distribution of static FLT-PET in a control participant and a patient with AML, (ii) CT-based anatomical FLT distribution in the patient with AML. B-D, The SUVs mean (SUVmean) and maximum (SUVmax) of specific regions. AML indicates acute myelogenous leukemia; CT, computed tomography; FLT, 3′-deoxy-3′[(18)F]-fluorothymidine; PET, positron emission tomography; SUV, standardized uptake value.

Figure 1A (i and ii) shows WB distributions of FLT-PET. Even with 80% blasts as measured by the marrow aspirate in the patient with AML, the FLT was inhomogeneously distributed, especially in the femur. Figure 1B-D shows SUVs of specific regions. The patient with AML has higher activity than the control participant. Overall, leukemic activity is relatively higher in the spine than in other skeletal regions. The difference between SUVmax and SUVmean is more pronounced in femoral regions than in spine regions. This result is potentially because of a large heterogeneity of SUV distribution in locally focused regions (Figure 1A [ii]). The site-specific nature of FLT activity will be monitored in future clinical trial.

**Figure 2.**
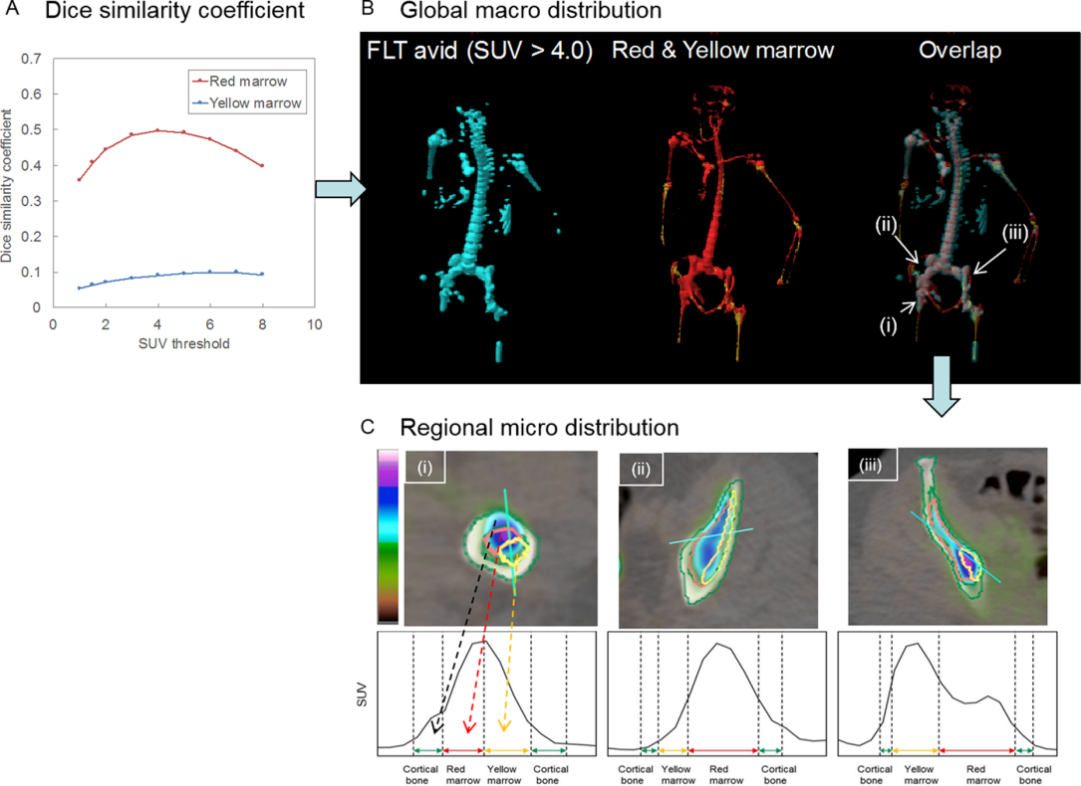
Comparison of FLT-based leukemia niche and DECT-based marrow regions. A, Dice similarity coefficient between FLT-avid regions and red or yellow marrow regions of a patient with AML. B, Global distribution of leukemia (FLT-avid [cyan], SUV threshold >4.0), red marrow (red), and yellow marrow (yellow) regions. C, Regional distribution of leukemia in 3 skeletal regions with structures of cortical bone (green), red marrow (red), and yellow marrow (yellow). AML indicates acute myelogenous leukemia; DECT, dual-energy computed tomography; FLT, 3′-deoxy-3′[(18)F]-fluorothymidine; SUV, standardized uptake value.

Figure 2A shows the DSC between the regions obtained by FLT-PET and DECT of the patient with AML. The maximum DSC value between RM regions and FLT-avid regions was 0.497, with the SUV threshold >4.0. On the other hand, the DSC value remains less than 0.1 between YM regions and FLT-avid regions. Figure 2B shows the distribution of FLT-avid regions (SUV threshold >4.0) and RM and YM regions. Figure 2C shows profiles of SUV in 3 local regions with structures of cortical bone, RM, and YM. This specific patient had predominantly high RM activity. At an older age, and due to treatment effect, the YM content increases heterogeneously as previously reported.^10^ Further detailed analysis (Figure 2C) reveals colonization of leukemia in the RM, YM, and endosteum, suggesting a potential association of leukemia disease with skeletal locations and local BME. To the best of our knowledge, this is the first report showing skeletal-wide preferential leukemia homing in the BME beyond the commonly known hematopoietic marrow niche. This method will facilitate investigating the potential association of the skeletal macroenvironment (eg, BM adipocytes) and leukemia relapse in future clinical studies.

**Figure 3.**
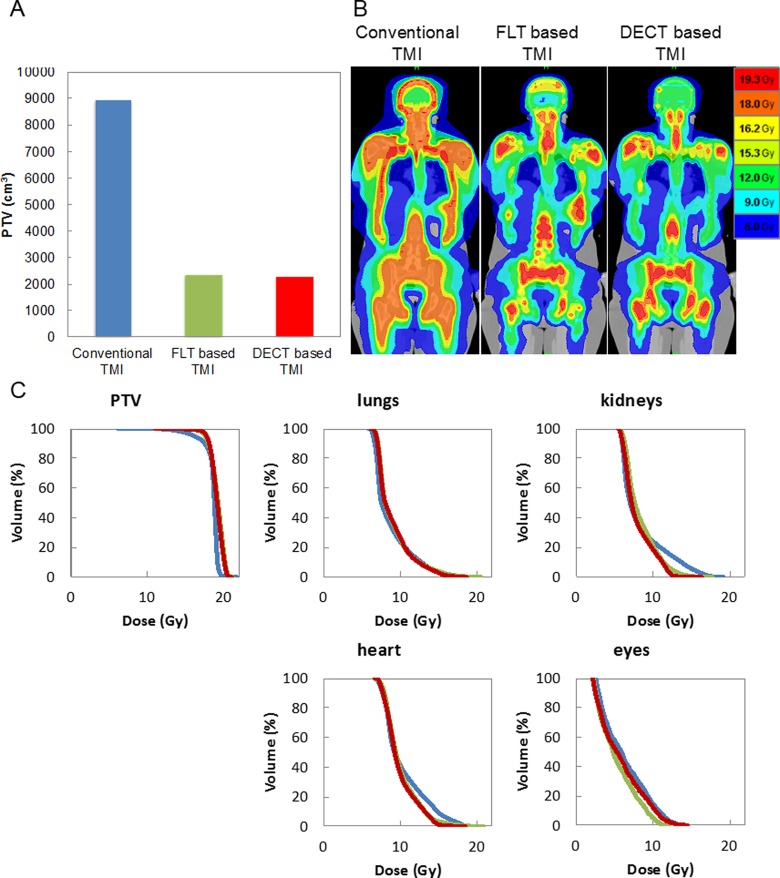
Comparison of conventional TMI, FLT-based TMI, and DECT-based TMI. A, Comparison of planning target volumes. B, Dose distributions of conventional, FLT-based, and DECT-based TMI plans. C, Dose–volume histograms of conventional (blue), FLT-based (green), and DECT-based (red) TMI plans. DECT indicates dual-energy computed tomography; FLT, 3′-deoxy-3′[(18)F]-fluorothymidine; TMI, total marrow irradiation.

**Figure 4.**
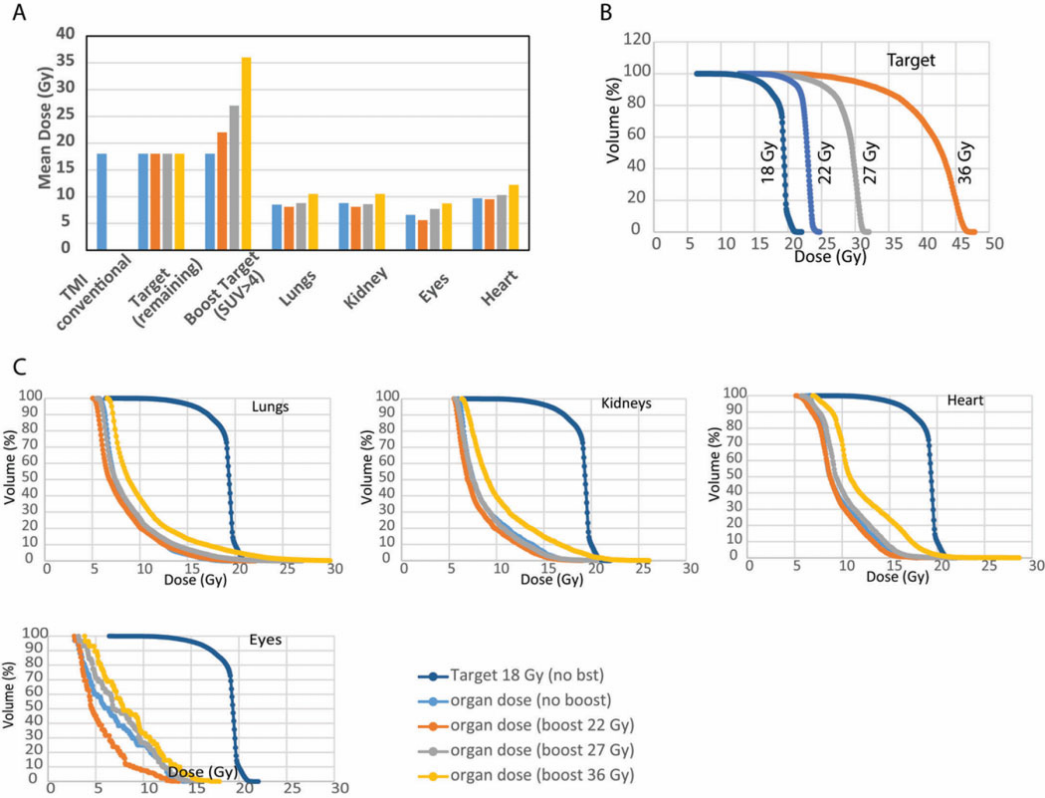
Assessment of stepwise dose escalation (boost) to FLT-avid (SUV >4) regions. A, Mean dose (Gy) comparisons of various targets and critical organs while increasing dose to FLT-avid region. B. The DVHs coverage of FLT-avid (boost target) at different dose levels (18, 22, 27, and 36 Gy). The PTV target (defined as 18 Gy) represents the entire skeleton for conventional TMI planning, and it represents the remaining skeletal region separated from the FLT-avid region for boost treatment. C, Changes in DVHs for critical organs (lungs, kidneys, heart, and eyes) when the FLT-avid dose is increased from 18 to 36 Gy (shown in B). DECT indicates dual-energy computed tomography; DVHs, dose–volume histograms; FLT, 3′-deoxy-3′[(18)F]-fluorothymidine; PTV, planning target volume; SUV, standardized uptake value; TMI, total marrow irradiation.

To investigate the therapeutic advantage of this multimodal imaging, treatment planning simulations were performed. In category I simulation, the primary PTV of FLT and DECT-based TMI was reduced to 26% and 25%, respectively, compared with conventional TMI (Figure 3A). Figure 3B shows the dose distribution of conventional, FLT, and DECT-based TMI plans, and Figure 3C shows corresponding dose–volume histograms (DVHs), suggesting that doses to critical organs could be reduced in FLT and DECT-based TMI plans. This approach presumably may be applied to kill more leukemic cells in targeted regions in comparison to conventional TMI, as well as reduce radiation to other BM sites to potentially preserve the BME. In category II, Figure 4 shows stepwise dose escalation to FLT-avid (SUV >4) regions, from 18 up to 36 Gy, while doses to critical organs were similar. The DVHs of FLT-avid regions (boost target) at different dose levels and associated DVHs for critical organs are shown in Figure 4B and C. At the highest dose escalation to boost target (100% increase), DVHs for lungs, kidneys, and heart show slight (∼10%) enhanced radiation exposure. However, these dose exposures to critical organs are still much less than the WB exposure of 12 to 13.2 Gy from commonly used myeloablative total body radiation conditioning regimens. Future clinical evaluation will be required to optimize boost dose while limiting organ toxicity in an appropriate population that may benefit. For example, Stein et al achieved radiation dose escalation to the entire skeletal target with increased survival benefit; however, relapses continue to be high.^14^ A further increase in dose to the skeletal system is not feasible due to increased dose to critical organs, which could lead to organ toxicities. Our simulation suggests potential dose enhancement to PET-avid regions to kill comparatively higher numbers of radiosensitive leukemia cells without increasing toxicities to critical organs. A heterogeneous distribution of AML in the skeletal system was reported to be associated with chemoresistance.^11^ To evaluate the impact of heterogeneous distribution and location of PET-positive regions on this dose escalation strategy, an observational clinical trial may be a necessary next step. As the CT scan is part of PET-CT imaging, integration of this process will be straightforward and cost-effective. However, one may need to verify marrow composition with a “gold standard” magnetic resonance imaging in some skeletal sites, as shown in our previous work.^15^ Because the low patient numbers herein pose a limitation, this method is being implemented with a larger patient population in a prospective clinical trial.

In conclusion, we developed a hybrid system of functional–anatomical–physiological imaging that offers the possibility of identifying the spatial distribution of leukemia on site-specific skeletal compositions. This method will facilitate the future clinical investigation of the role of the skeletal macro- and microenvironment in leukemia disease patterns and relapse. Additionally, FLT-PET-guided fTMI could offer an alternative option for further dose escalation strategies in a patient population with a high risk of relapse.
